# Surprising absence of association between flower surface microstructure and pollination system

**DOI:** 10.1111/plb.13071

**Published:** 2019-12-12

**Authors:** M. Kraaij, C. J. van der Kooi

**Affiliations:** ^1^ Groningen Institute for Evolutionary Life Sciences University of Groningen Groningen the Netherlands

**Keywords:** Colour, epidermal cones, grip, micro‐papillae, microstructure, pollination

## Abstract

- The epidermal cells of flowers come in different shapes and have different functions, but how they evolved remains largely unknown. Floral micro‐texture can provide tactile cues to insects, and increases in surface roughness by means of conical (papillose) epidermal cells may facilitate flower handling by landing insect pollinators. Whether flower microstructure correlates with pollination system remains unknown.
- Here, we investigate the floral epidermal microstructure in 29 (congeneric) species pairs with contrasting pollination system. We test whether flowers pollinated by bees and/or flies feature more structured, rougher surfaces than flowers pollinated by non‐landing moths or birds and flowers that self‐pollinate.
- In contrast with earlier studies, we find no correlation between epidermal microstructure and pollination system. The shape, cell height and roughness of floral epidermal cells varies among species, but is not correlated with pollinators at large. Intriguingly, however, we find that the upper (adaxial) flower surface that surrounds the reproductive organs and often constitutes the floral display is markedly more structured than the lower (abaxial) surface.
- We thus conclude that conical epidermal cells probably play a role in plant reproduction other than providing grip or tactile cues, such as increasing hydrophobicity or enhancing the visual signal.

## Graphical abstract

The shape and size of floral epidermal cells differs between different flower sides, but is not associated with pollinator guild across different genera.

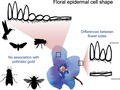

## Introduction

**Figure 1:**
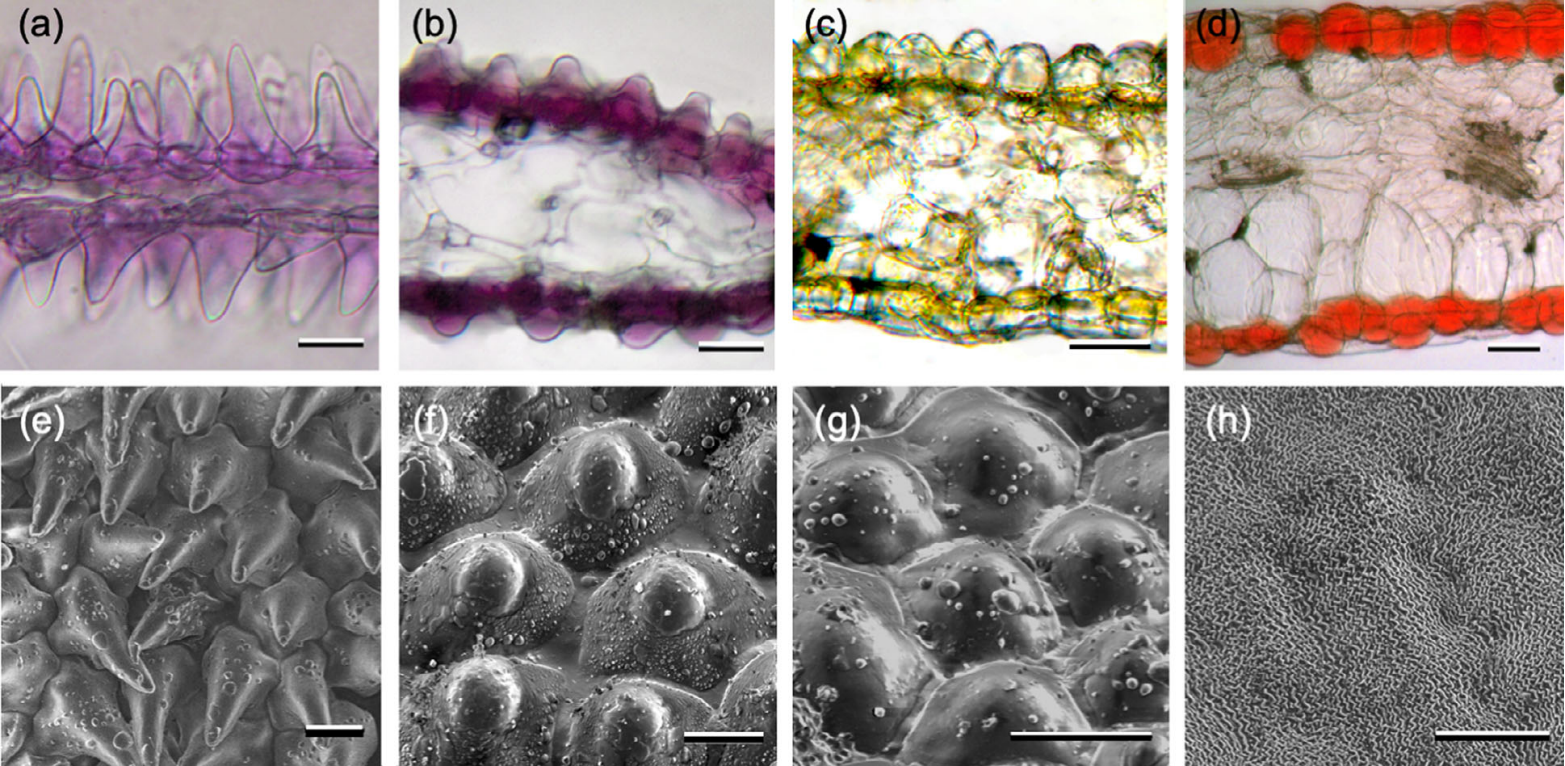
Variation in epidermal surface shape and structure of flowers. (a) *Solanum citrullifolium*, (b) *Aquilegia vulgaris*, (c) *Impatiens scabrida*, (d) *Phaseolus coccineus*, (e) *Solanum citrullifolium*, (f) *Impatiens sodenii*, (g) *Nicotiana bonariensis*, (h) *Clarkia breweri*. Scale bars: a, b, e–h: 20 μm, c, d: 50 μm.

The variety in shape and structure of flower surfaces and their function in terms of interactions between plants and insects has intrigued scientists for decades (*e.g*. Kay *et al*. 1981; Lee 2007; Whitney *et al*. 2011; Papiorek *et al*. 2014; van der Kooi *et al*. 2014; Ojeda *et al*. 2016). There are three types of epidermal cell shapes in flowers, distinguished by their shape. Flat cells are the least common type, but are characteristic of flowers in the buttercup genera *Ranunculus* and *Ficaria* (Ranunculaceae), contributing to their glossy appearance (Kay *et al*. 1981; Vignolini *et al*. 2012; van der Kooi *et al*. 2017). More common types of shapes are convex and cone‐shaped (Fig. 1). The epidermal cells of leaves and stems are generally flat, why then do flowers of so many species have cone‐shaped epidermal cells?

At least three non‐mutually exclusive hypotheses as to the functional significance of cone‐shaped epidermal cells have been proposed. First, the cone shape may contribute to the flower’s visual signal by focusing or scattering incident light. Modelling studies suggested that under perpendicular illumination, conical epidermal cells could act as micro‐lenses that focus incident light on the floral pigments (Gorton & Vogelmann 1996; Wilts *et al*. 2018), resulting in reflected light being more strongly filtered by pigments and thus generating stronger flower coloration. However, experimental studies with bees showed that presence or absence of cones does not change the flower’s salience as perceived by bees (Dyer *et al*. 2007). Further, given the often strongly varying illumination in nature and varying epidermal cell shape and spacing – even between neighbouring cells (Fig. 1; van der Kooi *et al*. 2014; Fritz *et al*. 2017), it remains unclear whether focusing effects play a biologically meaningful role in natural conditions (reviewed by van der Kooi *et al*. 2019a).

Second, the roughness of a flower surface may play a role in the flower’s hydrophobicity and self‐cleaning. Surface roughness, by means of epidermal cones or cuticle striations, reduces the contact area – and thus adhesion – of water droplets and the flower, causing water to roll of the flower (Neinhuis & Barthlott 1997; Barthlott & Neinhuis 1997; Taneda *et al*. 2015). Indeed, both modelling and experimental approaches showed that epidermal cell shape (in combination with a structured cuticle) determines the wettability of flowers (Taneda *et al*. 2015).

Third, the flower surface may provide a tactile cue and/or mediate the amount of grip an insect has upon landing on the flower. A tactile role of flower surfaces was first shown by Kevan & Lane (1985), who demonstrated that bees can discriminate flowers based on surface microstructure. Further, the minute claws and hairs at the tarsi of flower‐visiting insects indeed adhere better to rough flower‐like surfaces (Voigt *et al*. 2012; Bräuer *et al*. 2017). Studies using isogenic *Antirrhinum majus* lines and flower surface replicas that differed solely in epidermal cell shape, confirmed that bees can discriminate flowers based on touch alone and that epidermal cones may provide grip to bees that visit the flowers (Whitney *et al*. 2009). The importance of microstructures in providing grip to visiting insects is further likely to increase with verticality of the floral display.

If conical epidermal cells provide grip to landing insects then, conversely, their absence could be an ‘anti‐bee’ adaptation (sensu Castellanos *et al*. 2004). For example, (nearly) flat and smooth epidermal cells may hamper flower handling by floral antagonists such as nectar‐robbing insects (Papiorek *et al*. 2014; Ojeda *et al*. 2017). The pitchers of some species of carnivorous plants (*e.g*. *Nepenthes*) feature flat epidermal cells that are indeed slippery, causing prey to slide into the pitcher and be digested (Gaume *et al*. 2002). By the same reasoning, there may be differences in surface structure between flower areas that are frequently touched by pollinating insects (*e.g.* the landing area or surfaces surrounding the nectaries and anthers) *versus* areas that will rarely be touched by pollinators, such as the lower (abaxial) side of the floral display.

Following the hypothesis that flower surfaces may provide grip and/or tactile cues to specific insect pollinators and having an unstructured surface can deter floral antagonists, we may expect epidermal surfaces to correlate with pollinator guild. Whereas bees and flies land on the flower to forage nectar and pollen, birds and hawkmoths do not land but hover in front of the flower while feeding. Some birds sit on a nearby branch while drinking nectar from the flower. Similarly, flowers that reproduce *via* uniparental reproduction (*e.g.* self‐pollination or asexual reproduction) will also not need to provide grip or tactile cues to landing insects, so surface structure may be one of the multiple (pollinator‐attracting) traits that degenerate in flowers of self‐pollinating plants (*e.g*. Goodwillie *et al*. 2010; Sicard & Lenhard 2011; Dart *et al*. 2012).

In this study, we investigate the evolution of flower surfaces by virtue of comparing closely related species with contrasting pollination or mating systems. We include 13 genera across nine angiosperm families, and in total compare the floral epidermal cell shape for 29 congeneric sister species pairs with vertically presented flowers that differ in pollination or mating system. We test whether (i) flower surfaces that are frequently touched and/or seen by insect pollinators are more structured (*i.e*. have higher and/or more pointy, conical epidermal cells) than areas that are invisible/inaccessible to pollinators, and whether (ii) surfaces of bee‐ and fly‐pollinated flowers will be more structured than species that are pollinated by hawkmoths, birds or *via* self‐fertilisation. We found that there is no large‐scale correlation of flower surface and pollinator guild or mating system, but that the adaxial surfaces of flowers are markedly more structured than abaxial surfaces, hinting at abiotic and/or visual effects as main drivers for flower surface evolution.

## Material and Methods

### Species used

Plant species pairs were chosen *via* a thorough literature research. We included species that were specialised in their pollinator guild inasmuch as that they are exclusively bee‐/fly‐, moth‐ or bird‐pollinated, not mixed. In choosing our study species, we followed the most effective pollinator principle and not the ‘pollination syndrome’ concept, because this has been proven to not hold for numerous plant groups (Ollerton *et al*. 2009; Funamoto & Ohashi 2017). For the same reason, we included only plant species for which actual pollinating species were reported, thus excluding cases for which only flower visitors were known. Although we realise that within each category a plant may be serviced by a wide array of different species (Waser *et al*. 1996), these pollinators have similar behaviour with respect to flower handling, that is, the plants are functionally specialised (sensu Ollerton *et al*. 2007). Species were classified as (obligate) selfer only when there was clear evidence to suggest that selfing was the main reproductive mode, because – as for the pollination syndrome concept – presence of a phenotypical trait that typically occurs in selfers need not be good evidence that the species is actually largely selfing (*e.g.* Lozada‐Gobilard *et al*. 2019). Phylogenetic relatedness was determined using recently published phylogenies (see Supplementary Information for information on pairs and references). We thus included 46 species of 13 genera in nine plant families that covered 29 species pairs pollinated by guilds with contrasting flower handling behaviour (Table 1). Flowers were taken from plants grown from seed in the University greenhouse (LD: 14 h:10 h, day:night temperature 22:20 °C) or in Botanical Gardens (see Supplementary Information for details on sources).

**Table 1:** Summary of the taxa and pollinator mode included. Details regarding species pairs, pollinator guild/mating system and references are provided in the supplementary data file.

family	genus	comparison groups (number of pairs)
Acanthaceae	*Ruellia*	Bee‐bird (1)
Balsaminaceae	*Impatiens*	Bee‐bird (4), bee‐moth (2)
Campanulaceae	*Lobelia*	Bee‐bird (1)
	*Hippobroma* a	Bee‐moth (1)
Caryophyllaceae	*Silene*	Bee‐bird (1), bee‐moth (1), outcrossing‐selfing (1)
Lamiaceae	*Salvia*	Bee‐bird (2)
Onagraceae	*Clarkia*	Bee‐moth (1)
	*Oenothera*	Outcrossing‐selfing/asexual (1)
Phrymaceae	*Mimulus*	Bee‐bird (1), bee‐moth (3), outcrossing‐selfing (1)
Polemoniaceae	*Leptosiphon*	Outcrossing‐selfing (1)
Solanaceae	*Nicotiana*	Bee‐bird (2), bee‐moth (1)
	*Petunia*	Bee‐bird (1), bee‐moth (1)
	*Solanum*	Outcrossing‐selfing (2)

a All species pair comparisons are within one genus, except moth‐pollinated *Hippobroma longiflora*, which was compared with the closely related bee‐pollinated *Lobelia siphilitica*.

### Investigation of flower surfaces

We first compared the adaxial and abaxial surfaces of 12 bee‐/fly‐pollinated species. For the comparison between pollinator guilds and mating systems, we compared the adaxial sides, for in the studied species the adaxial surface will be seen and touched by pollinators. The surface of the floral organ that constitutes the visual display (generally the petal) was examined regardless of its colour (pattern) using dental impression material (Provil Novo Light, Heraeus Kulzer, Hanau, Germany) as per van der Kooi *et al*. (2014). Immediately after picking the flower, it was pressed into the freshly prepared dental impression material that solidifies within minutes. The mould was subsequently filled with transparent nail polish (base coat, Hema, the Netherlands), creating a positive surface replica, *i.e*. a cast. The casts were examined under a microscope (Nikon Diaphot 300) with a 40× objective. The surface structure of the whole floral organ was examined and the most dominant structure (generally that on the central/distal area) was photographed with a Nikon D70 camera. Epidermal cell shape dimensions (below) were measured in Fiji (ImageJ, NIH). To avoid observer bias, the flower surfaces were observed and measured by an observer (MK) who had no prior knowledge of the pollinator, *i.e*. the observer was ‘blind’ (Holman *et al*. 2015; Kardish *et al*. 2015). Transverse sections of fresh flowers shown in Fig. 1 were obtained by photographing a transverse section of a piece of flower embedded in 6% agarose (for details, see van der Kooi & Stavenga 2019).

### Analyses of surface parameters

**Figure 2:**
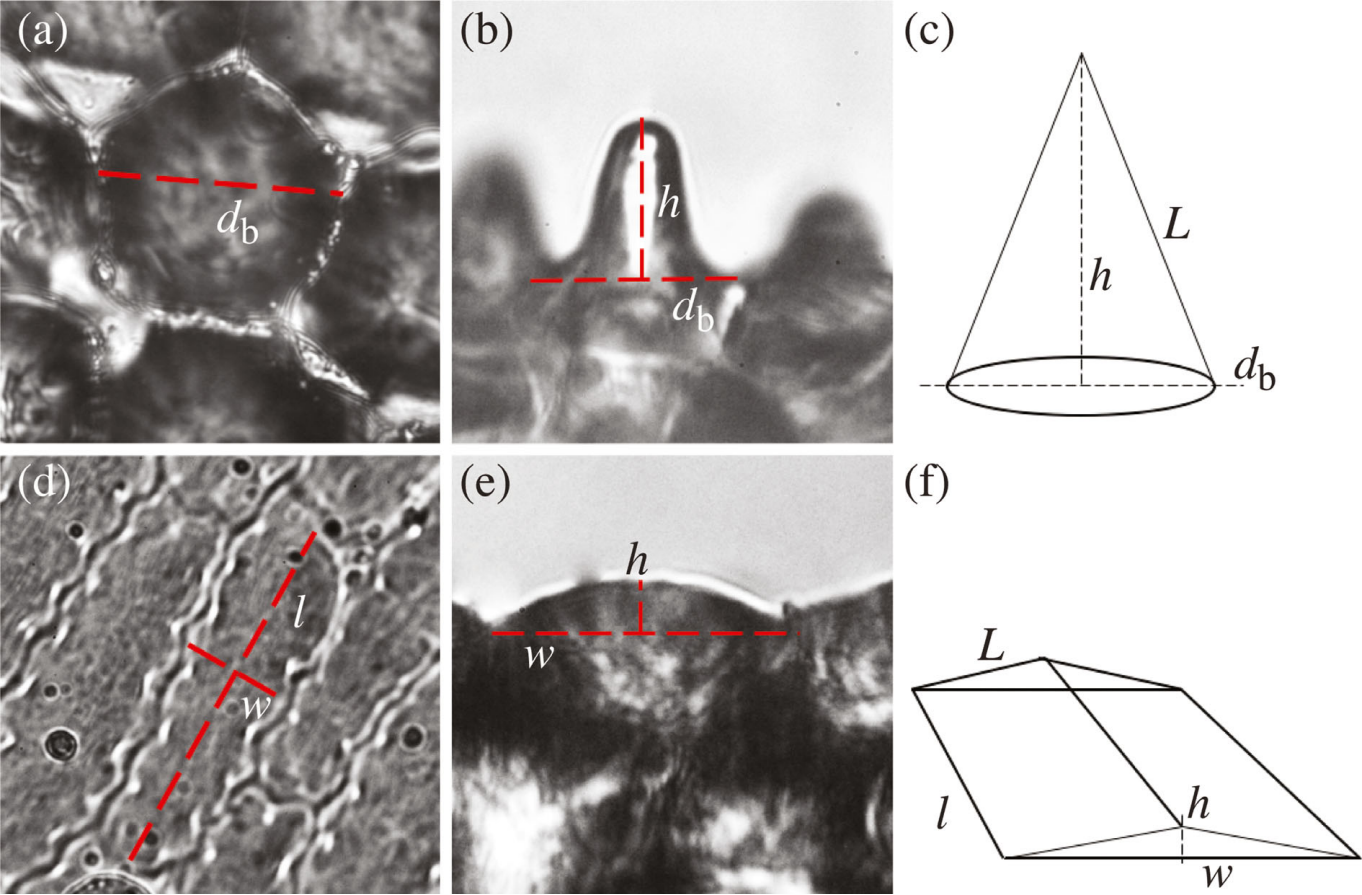
Measuring different types of epidermal cell shapes. Convex (or dome‐shaped) cells generally have a rectangular/elongated base cell shape, whereas conical epidermal cells have a circular/hexagonal base cell shape. Left column: shape and measurement of conical epidermal cells; right column: shape and measurement of convex cells. (a,d) top view, (b,e) side view, (c,f): measurements and calculations of different cells shapes. The roughness index was calculated as the ratio of the 3D lateral surface of the cell to the ground plane of the cell’s base (see Methods) db: diameter of cell base, l: cell base length, w: cell base width, h: protruding height, L: long side length.

For cells with a round or (quasi‐)hexagonal base, we measured the diameter of the cell’s base, and for cells with a rectangular base, we measured cell length and width (Fig. 2a, b). The protruding height was measured similarly for both types of epidermal cell (Fig. 2c, d). Preferably, we measured the floral surfaces of three individual plants per species, for which we succeeded in 34 out of 46 species (74%), but for nine species we could sample only two individuals and for three species only one. The approximate roughness of a surface was calculated *via* a ‘roughness index’, which is the ratio of the lateral, 3D surface area to the projected, geometric surface area of the cell’s base (following Aideo & Mohanta 2018). For cone‐shaped cells, the lateral surface area of the cone is described by:

$$S_{l}=L\times \;0.5\;\times \;d_{b}\;\times \;\pi$$

and the cell base size by:

$$C_{b}={\left(0.5\;\times \;d_{b}\right)}^{2}\times \;\pi$$

with *L* being the cone’s long side and *d*_b_ the cell’s base diameter (see Fig. 2e). For rectangular cells, the lateral surface area was calculated as:

$$S_{l}=2\times \;l\;\times L$$

and the cell base size as:

$$C_{b}=l\times w$$

with *l*, *L* and *w* being the base length, the long side length and width of the cells, respectively (see Fig. 2f). The roughness index is then the ratio of the lateral surface to the ground plane:

$$\text{RI}=S_{l}/C_{b}$$

For the exemplary cases shown in Fig. 1a–d, mean cell widths are 27, 22, 32 and 44 μm, respectively, and mean cell heights (measured as in Fig. 2c) are 34, 13, 10, 8 μm, respectively. The calculated roughness indices for these species then are 2.7, 1.6, 1.2 and 1.1, respectively, which is in line with the differences in roughness seen in Fig. 1a–d.

Statistical significance was tested *via* a parametric bootstrap test, using the *pbkrtest* package in R (R Foundation for Statistical Computing, Vienna, Austria). In the linear models, species was nested within genus as a random effect when testing the effect of flower side, and pair was nested within genus as random effect when testing the effect of pollination system. A likelihood ratio test (LRT) was used to see whether a model with the response variable (pollination system or flower side) fits better to the data than the same model without the response variable (1000 simulations). R script and data files are provided as Supporting Information.

## Results

**Figure 3:**
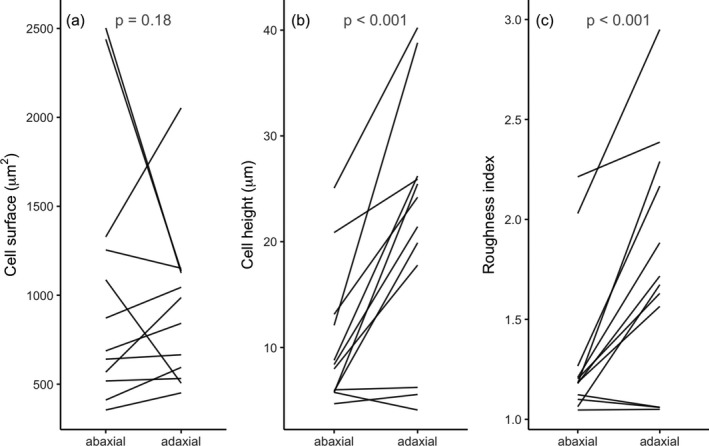
Floral epidermal cell shape for different flower sides. (a) Cell surface, (b) cell height, (c) roughness index. Each line connects the mean values of different sides of the flower. [Correction added on 10 January 2020 after first publication: the figure caption has been updated in this version.]

Our analysis of cell anatomy revealed significant differences between the adaxial and abaxial side of flowers (Fig. 3). The cell surface area is similar on both sides (LRT = 1.93, *P *=* *0.18), but the adaxial cell height is on average twice that of the abaxial side (LRT = 48.98, *P *<* *0.001). As cell height does not adequately capture overall surface curvature, we also calculated a roughness index, which is the ratio of the cell’s outer surface to that of the flat, projected cell surface area (Methods, Fig. 2). Analogous to the aspect ratio, the roughness index thus summarises the cell’s curvature relative to its ground plane. Indeed, the adaxial roughness index values are significantly larger than those of the abaxial surface (Fig. 3c; LRT = 44.3, *P *<* *0.001). In ten out of the 12 studied species, the adaxial epidermal cells are higher than the abaxial cells; in one species it is approximately the same and in one there is a small decrease in height; the roughness shows a similar effect (Fig. 3b, c). In the vast majority, cone‐shaped epidermal cells are thus particularly prominent at the adaxial side, and species with cone‐shaped cells on both sides (*e.g*. *Solanum citrullifolium*; Fig. 1a) are relatively rare.

**Figure 4:**
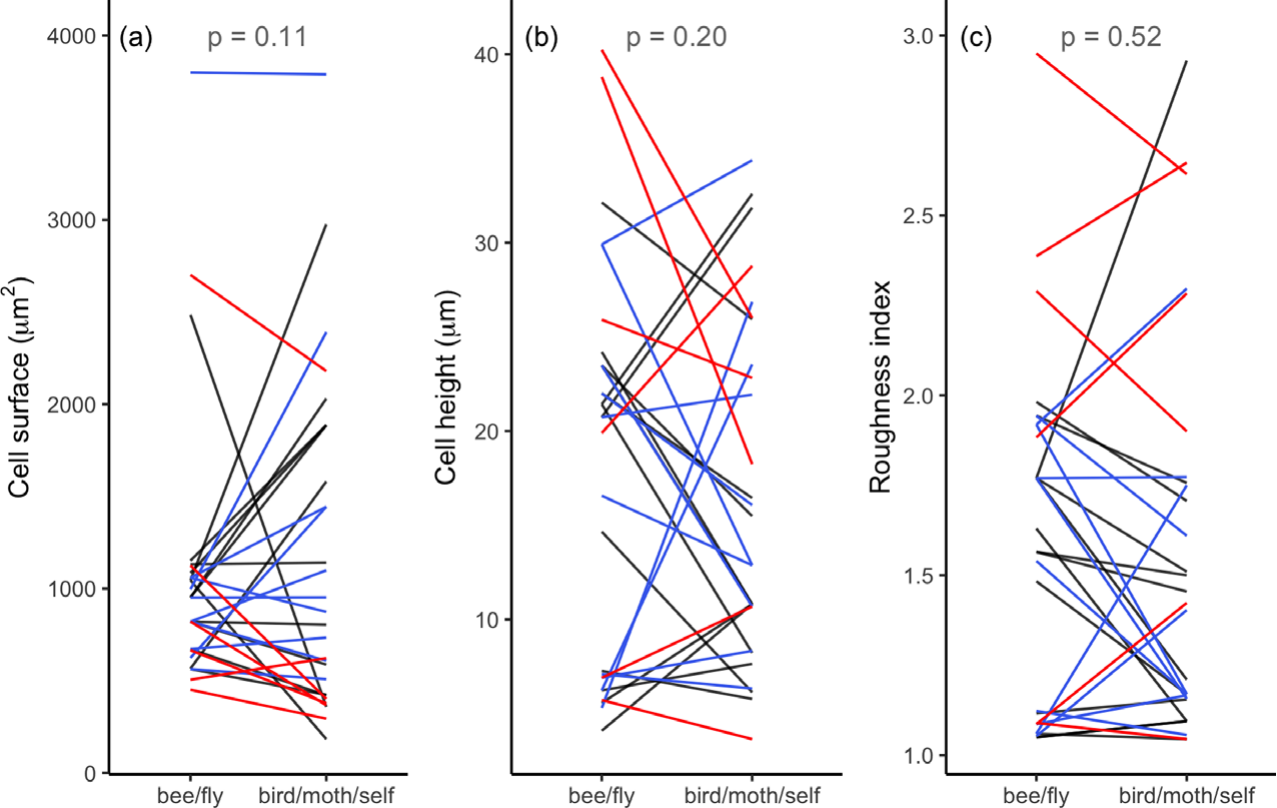
Floral epidermal cell shape in species pairs with different pollination systems. (a) Cell surface, (b) cell height, (c) roughness index. Each line connects the mean values of a species‐pair. Different colours represent different pollinator guilds or mating systems, bee/fly *versus* bird (black), moth (blue) and self‐pollination (red).

In contrast to observations between different sides of the flower, adaxial epidermal cell shape does not consistently differ between species with contrasting pollinators (Fig. 4; n = 29 species pairs, for all comparisons). Neither cell surface area (LRT = 2.57, *P *=* *0.11), cell height (LRT = 0.21, *P *=* *0.2) nor roughness index (LRT = 0.41, *P *=* *0.52) differs significantly between sister species. Also, when compared per pollinator guild, none are significantly different in epidermal cell height or roughness (Table S1).

In line with the reduction in floral display size commonly found in selfers (Goodwillie *et al*. 2010; Sicard & Lenhard 2011; Dart *et al*. 2012) is our observation that epidermal cell size is smaller in five out of six outcrossing–selfing species pairs (see the red curves in Fig. 4a). Floral epidermal cells of selfers are approximately 35% smaller in surface area than epidermal cells in related outcrossers, a difference that is marginally significant (*P *=* *0.036, n = 6 species pairs; Table S1). A similar trend is visible in epidermal cells of moth‐pollinated flowers, although this is not significant (*P *=* *0.072, n = 10 species pairs; Table S1).

## Discussion

### Flower surface does not correlate with pollination system

In our investigation of the microstructure of flowers with contrasting pollinators, we found no indication of large‐scale correlation of epidermal cell shape and pollination system (Fig. 4). The fact that the adaxial surface, which generally faces pollinators and is close to the reproductive organs, is markedly more structured than the abaxial surface (Fig. 3) suggests, however, that surface roughness fulfils a biologically meaningful function. Further, there is an effect of mating system inasmuch as that selfing plants have smaller epidermal cells than their outcrossing relatives (Fig. 4a; Table S1). This indicates that the reduction in floral display size commonly found in selfers (Goodwillie *et al*. 2010; Sicard & Lenhard 2011; Dart *et al*. 2012) is due, at least partly, to a reduction in cell size. Whether the number of epidermal cells, as well as the size and number of interior cells, also decrease in selfers requires additional investigation.

The fact that we find no correlation of flower epidermal cell shape and pollinator is not because sister species have not (yet) diverged sufficiently to develop phenotypic differences or because our method is inadequate to detect a biological signal. Indeed, we found up to three‐fold differences in epidermal cell surface, height and roughness index between sister species, albeit in opposite directions (Fig. 4); hence the absence of an overall pattern. In addition, (genetic) studies corroborate that shape of floral epidermal cells is evolutionarily labile (Glover *et al*. 1998; van Houwelingen *et al*. 1998; Di Stilio *et al*. 2009; Ojeda *et al*. 2017). Even within species, floral epidermal cell shape can vary; for example, a recent study on conical epidermal cells of 32 (cultivated) *Vicia faba* lines showed that 13% of the lines featured deviant epidermal cell shapes (Bailes & Glover 2018).

Our study does not invalidate previous experiments on tactile or grip effects of flower surfaces (Kevan & Lane 1985; Whitney *et al*. 2009), yet it does suggest that providing grip and/or tactile cues are not the main function of epidermal cones, at least not across angiosperms broadly. Thus, other functions are expected to shape flower surface microstructure. Our findings deviate from some earlier studies. Papiorek *et al *(2014) reported differences in epidermal cell shape of 58 species with bird or bee pollination; however, as they compared largely unrelated species, the observed phenotypic differences may be due to, for example, phylogenetic effects rather than pollinator guild. Ojeda *et al*. (2016) compared insect‐ and bird‐pollinated flowers and found that a transition to bird pollination confers a loss in epidermal cones. The discrepancy with our results may be explained by the fact that their sampling included relatively few species pairs (five comparison groups) and/or was based on categorical classification of cell shape. Our results dovetail those recently reported in two species of Nymphaeaceae with contrasting pollinators but no difference in flower surface (Coiro & Barone Lumaga 2018), and with those in a study on surfaces of 11 (unrelated) plant species with different pollinators (Costa *et al*. 2017).

It remains unknown how striations in the flower’s cuticle contribute to the mechanical interaction with pollinators. In the studied species, the frequent lack of cuticle structure precluded analysis of whether cuticle differences, if any, are linked to specific pollinators. Deeper species sampling would help to elucidate the importance of phylogeny on flower surface evolution. Additionally, in the systems so far considered, bird pollination was derived from bee/fly pollination and not *vice versa*, reflecting the asymmetry in the direction of transitions between insect and bird pollination found globally (Thomson & Wilson 2008; Barrett 2013). It remains to be investigated whether transitions from bird to bee pollination yield a systematic difference in epidermal cell shape, although we consider that unlikely.

### Adaxial flower side markedly more structured than abaxial side

The marked differences between the adaxial and abaxial surfaces (Fig. 3), combined with our observation that epidermal cell shape does not correlate with pollinator guild, suggest that cone‐shaped epidermal cells occur for reasons other than providing grip. Adaxial surfaces generally surround the reproductive organs, at least in the studied species, and having cone‐shaped cells may increase water repellence and even self‐cleaning of the flower (Neinhuis & Barthlott 1997; Barthlott & Neinhuis 1997; Watanabe‐Taneda & Taneda 2019), which could benefit flower visibility and longevity, and ultimately the plant’s reproductive success. Cone‐shaped epidermal cells could also modify the reflection of light by reducing the surface gloss and thus create a uniform visual signal that is well visible to pollinators approaching from different angles (van der Kooi *et al*. 2019a). Similar cone‐like structures were found to reduce surface gloss of some animal organs, such as moth eyes and snake skin (Stavenga *et al*. 2006; Spinner *et al*. 2013). Further, a higher surface roughness means an increase in surface area, which may lead to changes in transpiration rates (Buschhaus *et al*. 2015), flower temperature (van der Kooi *et al*. 2019b) and scent emission (Effmert *et al*. 2005).

The asymmetry in epidermal cell shape between adaxial and abaxial flower sides may be linked with the pigmentary aspects of the flower’s visual signal. Many species that have flowers with conical epidermal cells have pigments that occur in the epidermal cells only *(e.g*. flavonoids and anthocyanins; Kay *et al*. 1981; Grotewold 2006; Lee 2007; van der Kooi* et al.* 2016). Anthocyanin‐pigmented flowers often feature clear differences in pigmentation between abaxial and adaxial flower sides, resulting in only the pigmented side being strongly coloured, creating marked differences in coloration between the different sides (Stavenga & van der Kooi 2016; van der Kooi *et al*. 2016). Asymmetry in flower side coloration can be an efficient way of filtering reflected light with relatively little pigment (van der Kooi *et al*. 2016), and having only one coloured side can reduce conspicuousness to floral antagonists when the flower is closed (Kemp & Ellis 2019). Whether (asymmetry in) epidermal cell shape and pigmentation are indeed coupled requires further anatomical and optical investigation. Of particular interest would be to study the surfaces of species with hanging, pendant flowers where the abaxial flower side constitutes the visual signal.

## Author contribution

CJvdK designed the study, both authors collected the data, MK made the photographs and performed measurements, both authors performed the analyses and CJvdK wrote the manuscript. Both authors approved the final version of the manuscript.

## Supporting information


**Table S1.** P‐values for different comparisons. The p‐values obtained for the different sublevels were Bonferroni corrected for multiple testing.
**Data S1.** Information on species‐pairs, data file and R script.
