# Supplementary material for: Surprising absence of association between flower surface microstructure and pollination system
Source: Plant Biol (Stuttg). 2019 Dec 12;22(2):177–83. doi: 10.1111/plb.13071 (PMC7064994; doi:10.1111/plb.13071)
Supplement: Supplementary file 1 — Table S1. P‐values for different comparisons. The p‐values obtained for the different sublevels were Bonferroni corrected for multiple testing. Data S1. Information on species‐pairs, data file and R script. [file PLB-22-177-s001.zip › plb13071-sup-0001-TableS1.docx]

Table S1. P-values for different comparisons. The p-values obtained for the different sublevels were Bonferroni corrected for multiple testing.

| Pollinator comparison group | Cell surface | Cell height | Roughness index |
| --- | --- | --- | --- |
| Bee/fly-bird | 1 | 0.315 | 1 |
| Bee/fly-moth | 0.072 | 1 | 1 |
| Outcrossing-selfing | **0.036** | 1 | 1 |
