# Supplementary material for: Surprising absence of association between flower surface microstructure and pollination system
Source: Plant Biol (Stuttg). 2019 Dec 12;22(2):177–83. doi: 10.1111/plb.13071 (PMC7064994; doi:10.1111/plb.13071)
Supplement: Supplementary file 1 — Table S1. P‐values for different comparisons. The p‐values obtained for the different sublevels were Bonferroni corrected for multiple testing. Data S1. Information on species‐pairs, data file and R script. [file PLB-22-177-s001.zip › plb13071-sup-0007-Supinfo.docx]

Floral epidermal cell shape varies between species and sides of the flower, but is not associated with pollination system.
